# Tanyu Tongzhi Decoction Improves Cardiac Function by Inhibiting Platelet Activation and Alleviating Coronary Microthrombosis for Coronary Heart Disease Mice

**DOI:** 10.3390/ph19060823

**Published:** 2026-05-24

**Authors:** Ying Yang, Xiang Li, Danli Tang, Chengze Li, Sijia Wu, Yingying Li, Tong Lei, Wenjing Zong, Huamin Zhang

**Affiliations:** 1 Institute of Basic Theory for Chinese Medicine, China Academy of Chinese Medical Sciences, Beijing 100700, China; yangyingwac@163.com (Y.Y.);; 2 Experimental Research Centre of China Academy of Chinese Medical Sciences, Beijing 100700, China; 3 Institute of Chinese Materia Medica, China Academy of Chinese Medical Sciences, Beijing 100700, China

**Keywords:** coronary heart disease, multi-omics, platelet activation, network pharmacology, traditional Chinese medicine

## Abstract

**Background:** Coronary heart disease (CHD) has a high global disease burden. According to traditional Chinese medicine theory, the main syndrome type of CHD is the syndrome of intermingled phlegm and blood stasis (SI-GPBS). Tanyu Tongzhi Decoction (TYTZD) exerts clear cardioprotective effects on CHD patients with SI-GPBS, while its specific regulatory mechanism remains unclear. **Methods:** Clinical serum proteomics and network pharmacology were used to screen key targets and pathways for CHD with SI-GPBS. An APOE^−/−^ mouse model of CHD complicated with SI-GPBS was established and treated with TYTZD. Transcriptomics, proteomics and WGCNA were combined to screen core genes, with Western blotting, immunofluorescence, co-localization analysis and Carstairs staining for target verification and observation of coronary microthrombosis and endothelial injury. **Results:** A total of 754 differentially expressed proteins were identified in CHD patients with SI-GPBS, significantly enriched in the platelet activation pathway, with ITGA2B as the upregulated core hub protein. Network pharmacology found 94 active ingredients and 144 therapeutic targets of TYTZD for CHD with SI-GPBS, and key components bound well with ITGA2B. In APOE^−/−^ mice with SI-GPBS, TYTZD improved cardiac function, reduced blood lipids, myocardial enzymes, aortic lipid deposition and myocardial damage, downregulated ITGA2B, F2RL2, FGA and FGB, inhibited integrin αIIbβ3 signaling, restrained endothelial activation and reduced coronary microthrombosis. **Conclusions:** TYTZD treats CHD with SI-GPBS mainly by inhibiting platelet activation, improving endothelial dysfunction, and reducing coronary microthrombosis. This study provides experimental basis for TYTZD’s clinical application in CHD with SI-GPBS and new ideas for TCM syndrome–disease combination research.

## 1. Background

Coronary heart disease (CHD) is a cardiovascular disease with the highest morbidity and mortality globally and has emerged as a major public health concern that seriously threatens human health. In 2050, CHD will remain the leading cause of cardiovascular deaths [1]. Moreover, the age at onset has exhibited a significant downward trend in recent years. Modern medical research has confirmed that the occurrence and progression of CHD involve multiple interconnected pathological processes, including dyslipidemia, vascular endothelial dysfunction, oxidative stress, inflammatory cascade activation, cardiomyocyte apoptosis, and fibrosis, which together form a complex molecular regulatory network [2]. Despite substantial advances in clinical treatment in recent years, the long-term prognosis of patients with CHD remains suboptimal. Therefore, the development of safe and effective therapeutic strategies is of considerable clinical and social significance.

According to traditional Chinese medicine (TCM) theory, CHD falls into the categories of “Xiongbi” (chest bi-syndrome) and “Xintong” (heart pain). Syndrome of intermingled phlegm and blood stasis (SI-GPBS), a common pathological state characterized by lipid metabolic disturbance, chronic inflammation and microcirculation disorder in modern biomedical concepts, is recognized as the core pathogenesis of CHD in TCM theory [3]. Tanyu Tongzhi Decoction (TYTZD) is a TCM formula based on the classic prescription Gualou Xiebai Banxia Decoction (documented in Synopsis of the Golden Chamber by Zhang Zhongjing in the Eastern Han Dynasty), which has been traditionally used for treating SI-GPBS type CHD [4,5]. This formula is not a modern modified preparation but an optimized prescription inheriting the classic TCM theory of resolving phlegm and activating yang, combined with the therapeutic principle of promoting blood circulation to remove blood stasis. Tanyu Tongzhi Decoction (TYTZD) consists of six Chinese medicinal herbs: *Panax ginseng* C.A. Mey. (Ginseng Radix et Rhizoma), *Pinellia ternata* (Thunb.) Breit. (Pinelliae Rhizoma, processed), *Trichosanthes kirilowii* Maxim. (Trichosanthis Fructus), *Allium macrostemon* Bunge (Allii Macrostemonis Bulbus), *Paeonia lactiflora* Pall. (Paeoniae Radix Rubra), and *Ligusticum chuanxiong* Hort. (Chuanxiong Rhizoma). The botanical names of all herbal components in TYTZD were verified and confirmed with World Flora Online (www.worldfloraonline.org, accessed on 10 January 2026). This formula is derived from Gualou Xiebai Banxia Decoction, a classic prescription documented in Synopsis of the Golden Chamber (Jin Kui Yao Lue), written by Zhang Zhongjing, the Sage of Medicine of the Han Dynasty. Gualou Xiebai Banxia Decoction was formulated to activate yang to dissipate binds and resolve phlegm to broaden the chest [6]. Building on the core framework of resolving phlegm and activating yang in the original formula, TYTZD incorporates Chuanxiong Rhizoma and Paeoniae Radix Rubra, which promote blood circulation and remove blood stasis, as well as Ginseng Radix et Rhizoma, which tonifies qi and strengthens healthy qi. This evolution not only inherits the essence of resolving phlegm and activating yang from ancient prescriptions but also innovatively integrates the therapeutic principles of promoting blood circulation to remove blood stasis and tonifying qi to strengthen healthy qi. Consequently, the entire formula exerts synergistic effects of resolving phlegm and removing blood stasis, activating yang to relieve bi-syndrome, reducing turbidity and eliminating fat, and tonifying qi to strengthen healthy qi. Previous studies by our research group have demonstrated that TYTZD exerts anti-inflammatory, antioxidant, and lipid-lowering effects and exhibits significant cardioprotective effects of myocardial ischemia–reperfusion injury and atherosclerosis [5,7,8]. However, its specific mechanisms of action remain unclear.

Currently, the clinical treatment of CHD faces several challenges. Although statins can effectively regulate lipid levels, their long-term use may induce adverse reactions, such as liver injury and myotoxicity. Antiplatelet drugs carry a risk of bleeding, and some patients develop drug resistance [9]. Although vascular interventional therapy can rapidly recanalize stenotic blood vessels, issues, such as postoperative restenosis and the no-reflow phenomenon, have not been fundamentally resolved [10]. By contrast, the advantages of TCM in improving symptoms, reducing adverse reactions, and lowering recurrence rates have become increasingly prominent, making it an important component of the comprehensive treatment of CHD [11]. Owing to the characteristics of Chinese medicinal herbs, such as multi-component, multi-target and multi-pathway, there are considerable difficulties in comprehensively investigating their bioactive components and systematically elucidating their therapeutic mechanisms [12]. A multi-omics integration strategy can provide insight into TCM-related research. Systems biology approaches, such as transcriptomics and proteomics, combined with data-driven omics analysis techniques, can comprehensively decipher the complex interaction mechanisms between Chinese medicinal compounds and diseases at the molecular network level [13]. This study identified the key molecules of the disease from the serum proteomics of clinical CHD patients with SI-GPBS syndrome, constructed a corresponding mouse model using ApoE^−^/^−^ mice, ApoE is a key regulator of lipid metabolism, and ApoE^−^/^−^ mice spontaneously develop hyperlipidemia, atherosclerosis, and subsequent coronary heart disease under normal chow or high-fat diet conditions, which highly mimics the pathological process of human CHD. It also utilized multi-omics analysis to systematically explore the cardioprotective effects and molecular mechanism of TYTZD for CHD.

## 2. Results

### 2.1. Identification of Core Target in Platelet Activation-Related DEPs for CHD Patients

**Figure 1:**
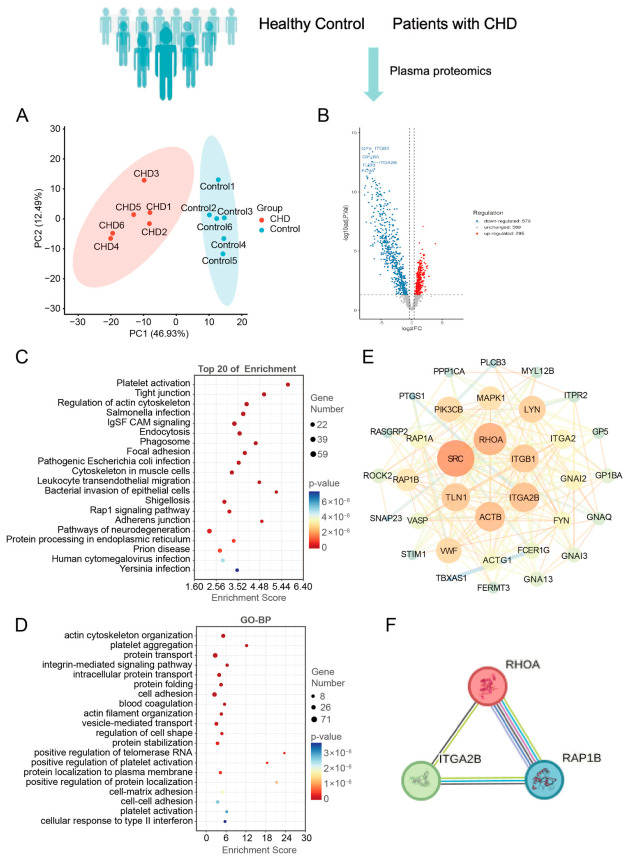
**Clinical proteome of CHD patients with SI-GPBS.** (**A**) PCA plots of the healthy control group and the coronary heart disease group. (**B**) Differential protein volcano plots between the healthy control group and the coronary heart disease group. (**C**,**D**) KEGG and GO–BP bubble chart of differential proteins. (**E**) PPI map of proteins related to platelet activation. (**F**) Core proteins related to platelet activation.

In order to identify the pathological targets of CHD with SI-GPBS, we conducted a clinical serum proteomics analysis of patients with CHD with SI-GPBS to identify the key molecules of the disease. Principal component analysis revealed significant intergroup differences between the TYTZD and model groups (Figure 1A). Compared with the healthy control group, 754 DEPs were identified in the CHD group (Figure 1B), which were mainly enriched in platelet activation, aggregation, and related signaling pathways based on KEGG and GO–BP enrichment analyses (Figure 1C,D). Thirty-four DEPs closely related to platelet function were used to construct a protein–protein interaction network, and the MCODE algorithm in Cytoscape identified RhoA, RAP1B, and ITGA2B as core node proteins with significantly upregulated expression in the CHD group (Figure 1E,F). ITGA2B, a terminal target of upstream regulators (including RhoA and RAP1B) and an activator of upstream pathways via outside-in reverse signaling, forms a positive feedback loop for platelet activation and acts as a key hub in maintaining platelet activation and thrombus stability, making it a potential therapeutic target for CHD.

### 2.2. TYTZD Regulates Platelet Activation in CHD with SI-GPBS

**Figure 2:**
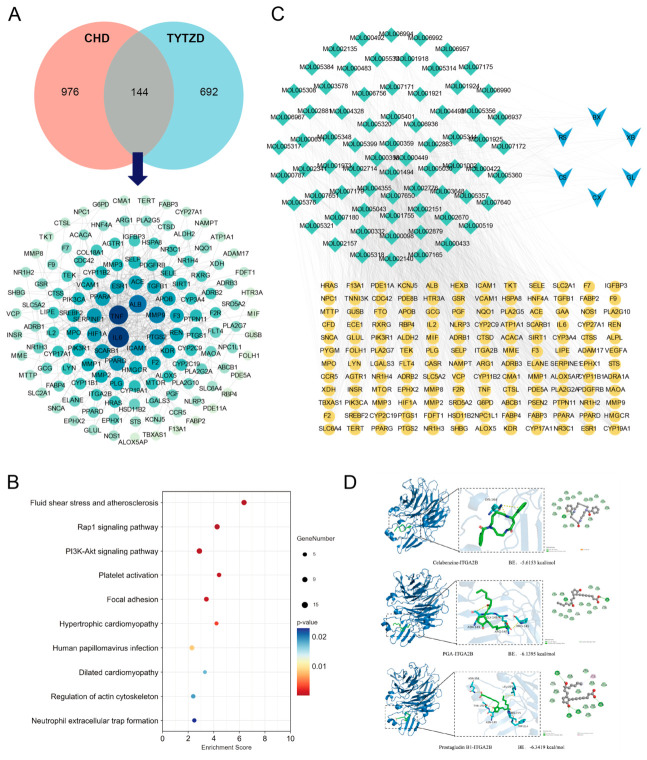
**Network pharmacology analysis revealed that the TYTZD exerts its anti-CHD effect by regulating platelet activation.** (**A**) PPI network diagram of drug–disease target intersections. (**B**) KEGG bubble diagram of drug–disease target intersections. (**C**) The “drug–component–target” network diagram. (**D**) Molecular docking schematic diagram of ITGA2B with Celabenzine, PGA1 and Prostaglandin B1.

Ninety-four active components of TYTZD with 836 target genes were identified in the Traditional Chinese Medicine Systems Pharmacology Database and Analysis Platform database. A total of 1120 CHD-related targets were retrieved from the GeneCards and Online Mendelian Inheritance in Man databases. The intersection of TYTZD targets with disease targets yielded 144 potential targets of TYTZD for CHD treatment (Figure 2A). KEGG enrichment analysis showed that, under TYTZD intervention, pathways, including fluid shear stress and atherosclerosis, the Rap1 signaling pathway, the PI3K–Akt signaling pathway, platelet activation, and neutrophil extracellular trap formation were significantly enriched (Figure 2B). These pathways are closely associated with the occurrence and progression of CHD, reflecting the multi-component, multi-target, and multi-pathway regulatory characteristics of the therapeutic effects of TYTZD on CHD. The active components corresponding to the potential therapeutic targets were compiled in Excel to construct an “component–target” network (Figure 2C). The active components targeting ITGA2B were Celabenzine, PGA1, and Prostaglandin B1. Molecular docking showed binding energies of −5.6, −6.1, and −6.3 kcal·mol^−1^, respectively (Figure 2D). Thus, TYTZD exerts its therapeutic effect on CHD by regulating platelet activation.

### 2.3. TYTZD Improves Cardiac Function, Ameliorates Myocardial Pathological Lesions, and Regulates Lipid Metabolism in CHD Mice

**Figure 3:**
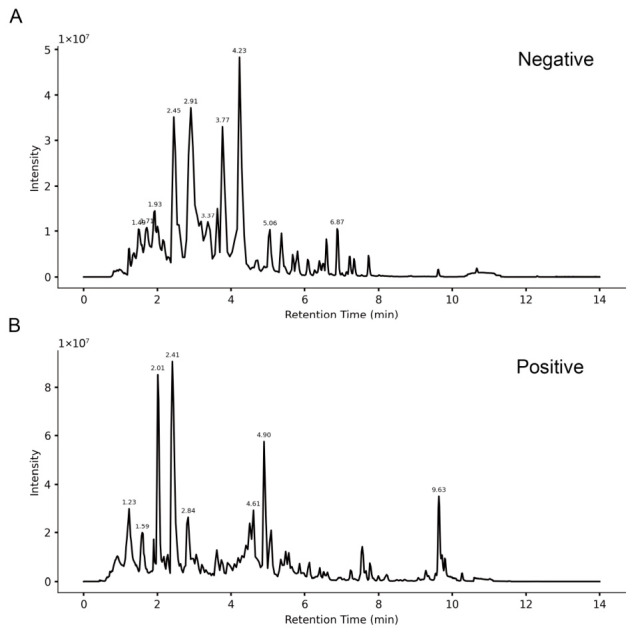
**Total ion chromatograms of UHPLC–MS/MS.** (**A**) Negative ion mode. (**B**) Positive ion mode.

**Figure 4:**
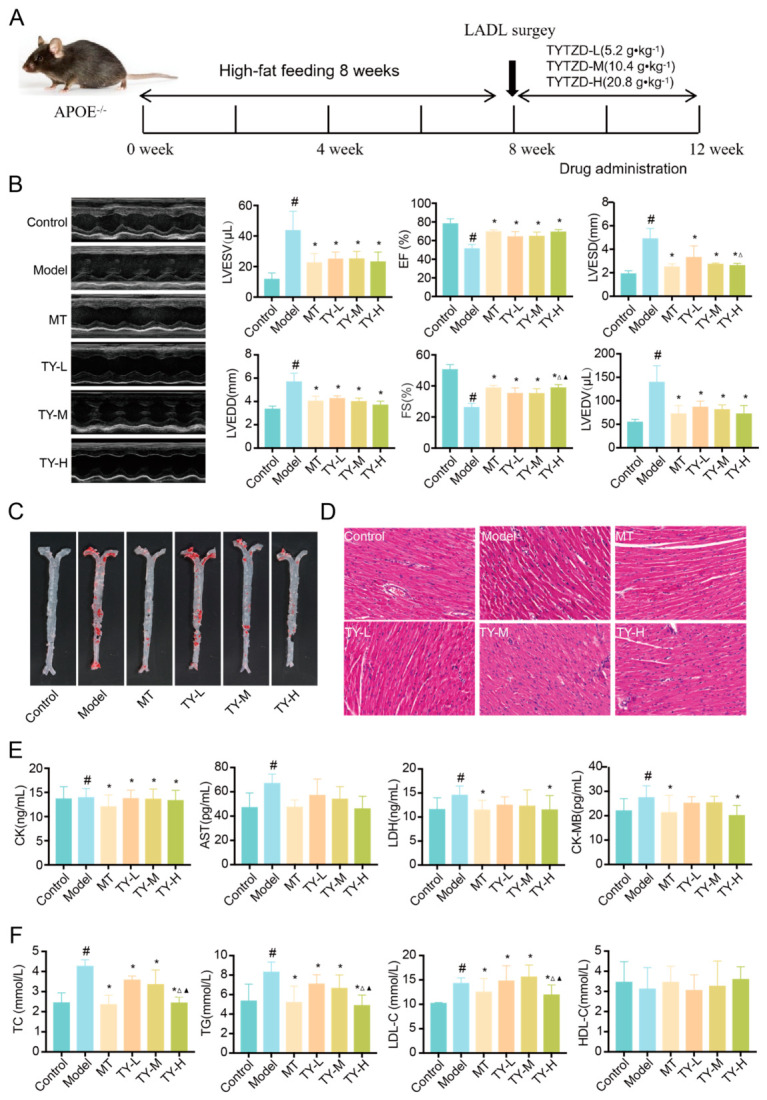
**Evaluation of the therapeutic effect of TYTZD on CHD mice with SI-GPBS.** (**A**). Animal flowchart (**B**). Cardiac function (EF%, FS%, LVESD, LVEDD, LVESV and LVESD) (**C**) Gross Oil Red staining of the aorta. (**D**) H&E staining (200×). (**E**) The levels of myocardial enzyme (CK, AST, LDH and CK-MB) (**F**). The levels of blood lipid indicators (TC, TG, HDL-C, LDL-C). The data are mean ± SD. * *p* < 0.05 compared to Model group. # *p* < 0.05 compared to Control group. In comparison to the TY-L group, ^△^ *p* < 0.05. In comparison to the TY-ML group, ^▲^ *p* < 0.05.

The total ion flow diagrams in the positive- and negative-ion scanning modes were collected separately (Figure 3). The contents of the following ten compounds detected in EZW were determined by UPLC–MS/MS: Paeoniflorin, Ferulate, Ginsenoside Re, Ginsenoside Rg1, Ginsenoside Rb1, Neoligustilide. The MRM parameters of these compounds in EZW are listed in Table 1. Cardiac function was significantly impaired in the model group, with reduced EF and FS and increased left ventricular end-systolic/diastolic volumes and left ventricular end-systolic/diastolic diameters (*p* < 0.05). Both MT and TYTZD improved cardiac function, with the high dose of TYTZD group (TYTZD-H) exhibiting the most pronounced effect (Figure 4B). Aortic Oil Red O staining revealed extensive lipid deposition and an increased plaque area in the model group, both of which were notably reduced by MT and TYTZD treatment (Figure 4D). Cardiac HE staining showed disorganized myocardial fibers, necrosis, and inflammatory infiltration in the model group, whereas MT and TYTZD improved fiber organization and reduced inflammation (Figure 4D). Serum CK, AST, LDH, and CK-MB levels were elevated in the model group (*p* < 0.05). MT and all TYTZD doses decreased CK levels (*p* < 0.05), whereas MT and TYTZD-H reduced LDH and CK-MB levels (*p* < 0.05) (Figure 4E). The model group exhibited increased TC, TG, and LDL-C levels and decreased HDL-C levels (*p* < 0.05). MT and all TYTZD doses lowered TC, TG, and LDL-C levels (*p* < 0.05), with TYTZD-H showing superior lipid-lowering effects compared with those of the low/medium dose of TYTZD groups (*p* < 0.05) (Figure 4F). In conclusion, TYTZD improved cardiac function and myocardial pathology and regulated blood lipid and myocardial enzyme levels in CHD mice, with TYTZD-H being the most effective. Therefore, samples from the TYTZD-H group were used for subsequent multi-omics analyses.

### 2.4. Multi-Omics Analyses Identify Core Targets and the Platelet Activation Pathway of TYTZD

**Figure 5:**
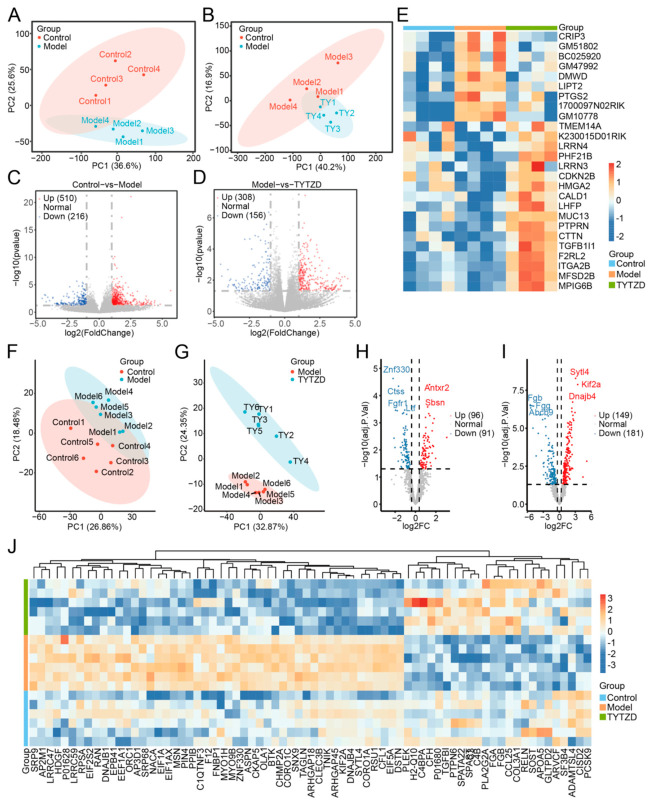
**Effect of TYTZD on transcriptome and proteome in mice with CHD.** (**A**,**B**) PCA score plot of the control group vs. the model group and the model group vs. the TYTZD group in Transcriptomics. (**C**,**D**) Differential protein volcano plots of the control group vs. the model group and the model group vs. the TYTZD group. (**E**) Heatmap of differential proteins reversed by TYTZD. (**F**,**G**) PCA score plot of the control group vs. the model group and the model group vs. the TYTZD group in Proteomics sequencing. (**H**,**I**) Differential gene volcano plots of the control group vs. the model group and the model group vs. the TYTZD group. (**J**) Heatmap of differential genes reversed by TYTZD.

**Figure 6:**
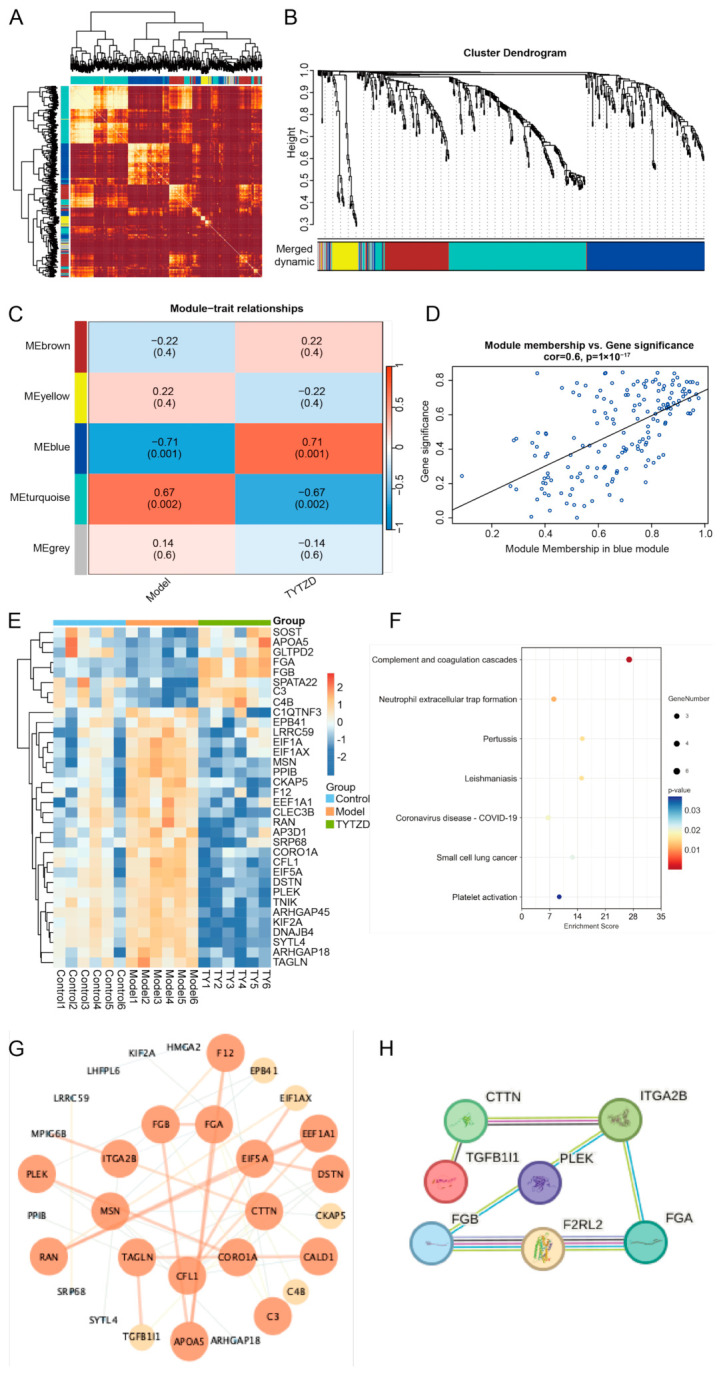
**Multi-omics Analyses Identify Core Targets in TYTZD for CHD.** (**A**) Differential protein volcano plots of the control group vs. the model group and the model group vs. the TYTZD group. (**B**) Dynamic Tree Cut and Module Assignment. (**C**) Module–Trait Relationship Heatmap. (**D**) Module Membership vs. Gene Significance Scatter Plot. (**E**) Heatmap of Proteins at the intersection of the green module that can be rescued by TYTZD Clustering. (**F**,**G**) PPI and KEGG bubble of 26 reversed DEGs and 35 co-expressed DEPs. (**H**) Core targets regulated by TYTZD.

To further elucidate the molecular mechanisms underlying TYTZD intervention in CHD phenotypes, transcriptomic and proteomic analysis was performed using mouse whole-blood samples. The results of PCA analysis showed significant differences in genes and proteins among the groups (Figure 5A,B,F–J). Comparison between the control and model groups identified 726 DEGs (510 upregulated and 216 downregulated) (Figure 5C), whereas 466 DEGs (308 upregulated and 158 downregulated) were detected in the TYTZD group compared with the model group (Figure 5D). Among the 726 DEGs identified between the control and CHD groups, 26 were reversed following TYTZD treatment (Figure 5E). Proteomic sequencing revealed 187 DEPs between the control and CHD groups (91 upregulated and 96 downregulated) (Figure 5H) and 330 DEPs (181 upregulated and 149 downregulated) in the TYTZD group compared with the model group (Figure 5I). Of the 187 DEPs identified between the control and CHD groups, 72 were reversed by TYTZD treatment (Figure 5G). A co-expression network was constructed based on DEGs from the CHD and TYTZD groups. After topological overlap matrix-based hierarchical clustering, dynamic pruning, and module merging, five co-expression modules were obtained. The blue module, containing 162 genes, was positively correlated with the treatment group (r = 0.60) (Figure 6A–D). Intersection of proteins in the blue module with TYTZD-reversed DEPs yielded 35 co-expressed DEPs (Figure 6E). By combining the 26 reversed DEGs and 35 co-expressed DEPs, 61 targets were obtained to construct a protein–protein interaction network (Figure 6F). Core targets, including F2RL2, FGA, FGB, PLEK, CTTN, TGFB1I1, and ITGA2B, were screened using the MCODE plugin in Cytoscape (Figure 6G). KEGG pathway enrichment analysis showed significant enrichment in signaling pathways, such as platelet activation (Figure 6H). These findings indicate that TYTZD may exert anti-CHD effects by regulating core targets, including F2RL2, FGA, FGB, PLEK, CTTN, TGFB1I1, and ITGA2B, thereby modulating the platelet activation pathway.

### 2.5. TYTZD Inhibits the Levels of Platelet Activation-Related Proteins

**Figure 7:**
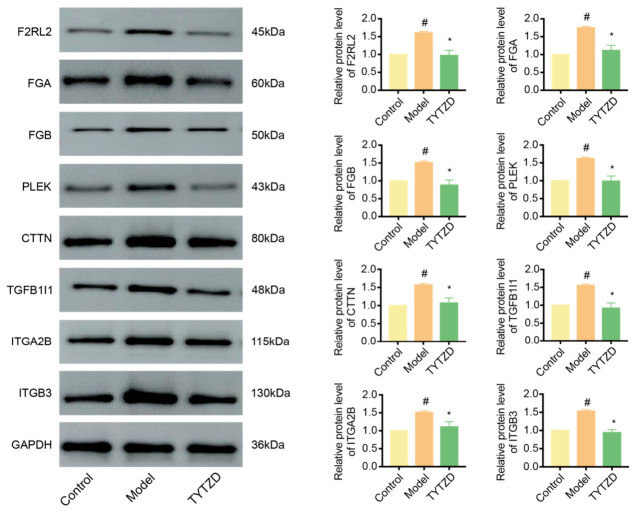
**TYTZD inhibits the expression of platelet activation-related proteins.** The protein expression of F2RL2, FGA, FGB, PLEK, CTTN, TGFB1I1, ITGA2B and ITGB3. The data are mean ± SD. * *p* < 0.05 compared to Model group. # *p* < 0.05 compared to Control group.

Platelet activation is a core link in arterial thrombosis, and F2RL2, FGA, FGB, PLEK, CTTN, TGFB1I1, and ITGA2B are key regulatory targets in platelet activation. Integrin αIIbβ3 is composed of the αIIb subunit encoded by ITGA2B and the β3 subunit encoded by ITGB3. Because it is the most abundantly expressed integrin on the platelet membrane, it is a hallmark of platelet activation [14]. The results showed that, compared with the control group, the protein expression levels of ITGA2B and ITGB3 were significantly increased in the model group (*p* < 0.05), whereas TYTZD reduced the protein expression of ITGA2B and ITGB3 (*p* < 0.05), thereby suppressing platelet activation. *F2RL2*, the gene encoding the platelet surface thrombin receptor PAR3, is an upstream initiator of the inside-out signaling pathway. PLEK encodes an important intracellular signal adaptor protein in platelets, acting as a core intermediate transduction molecule connecting upstream signals from F2RL2 to αIIbβ3. TYTZD downregulated the protein expression of F2RL2 and PLEK in the myocardial tissue of model mice (*p* < 0.05), thereby inhibiting integrin αIIbβ3 activation. Fibrinogen encoded by FGA and FGB is the core ligand for the adhesive function of αIIbβ3. TGFB1I1, as a midstream molecule in this pathway, can amplify αIIbβ3-mediated activation signals and enhance platelet adhesion. CTTN is a key downstream molecule involved in cytoskeletal rearrangement. It is activated by amplified signals and regulates actin reorganization to support morphological changes in platelets and irreversible aggregation. These three molecules synergistically ensure the stable formation of αIIbβ3-mediated thrombi. The results of this study showed that the protein expression levels of FGA, FGB, TGFB1I1, and CTTN in the myocardial tissue of model mice were significantly increased, whereas TYTZD intervention significantly reduced their protein expression levels (Figure 7), thereby inhibiting thrombosis induced by αIIbβ3 activation.

### 2.6. TYTZD Inhibits Myocardial Thrombosis and Endothelial Cell Vascular Activation in CHD Mice

**Figure 8:**
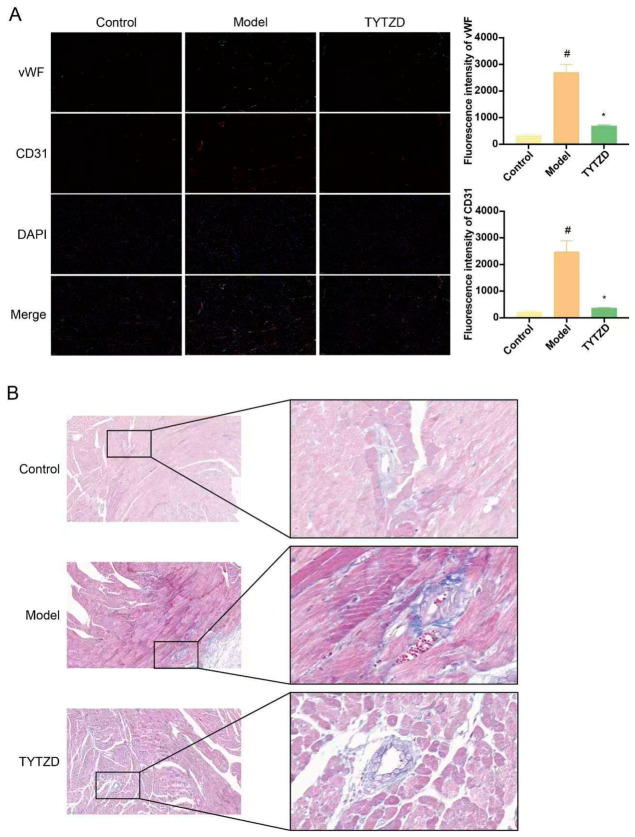
**Carstairs staining of Myocardial and endothelial cell angiogenesis.** (**A**) The double immunofluorescence labeling analysis showed the expression of vWF and CD31. (**B**) Carstairs staining of Myocardial (900×). The data are mean ± SD. * *p* < 0.05 compared to Model group. # *p* < 0.05 compared to Control group.

Endothelial cell injury and dysfunction are key initiating factors that trigger platelet activation and thrombosis. To further explore the regulatory effect of TYTZD on myocardial vascular endothelial cells in CHD mice, immunofluorescence co-staining for CD31 and VWF was performed on mouse cardiac tissue sections. The results showed that, compared with the control group, the CD41^+^vWF^+^ double-positive signal was significantly enhanced in the myocardial tissue of the model group. TYTZD intervention significantly downregulated CD31 and vWF expression (Figure 8A), indicating that TYTZD effectively inhibited abnormal activation of myocardial vascular endothelial cells in CHD mice. Cardiac sections from mice were subjected to pathological analysis to better characterize the morphology of coronary microthrombi. Carstairs staining results demonstrated that coronary thrombi in the model group were composed predominantly of dense fibrinogen and platelets, whereas TYTZD treatment reduced fibrinogen content within the thrombi, with only limited aggregation of platelets and blood cells (Figure 8B).

## 3. Discussion

CHD is a cardiovascular disorder with high morbidity and mortality. Clinical studies have demonstrated that abnormal platelet activation-induced adhesion, aggregation, and thrombosis can directly lead to coronary artery stenosis or occlusion, thereby triggering myocardial ischemia and necrosis. Therefore, targeted regulation of the platelet activation pathway has become a potential core strategy for CHD treatment [15]. However, single-target drugs often fail to address the intertwined pathological networks involved in platelet activation, vascular endothelial injury, and lipid metabolism disorders [16]. By contrast, Chinese herbal compound prescriptions may exhibit unique value in the treatment of complex diseases owing to the synergistic regulatory advantages of multiple components, targets, and pathways [17]. By integrating multi-dimensional evidence from clinical proteomics, network pharmacology, and animal experiments, we systematically investigated the anti-CHD mechanisms of TYTZD. The findings indicate that its core mechanism lies in the synergistic actions of multiple components targeting and regulating the platelet activation signaling network centered on integrin αIIbβ3, thereby inhibiting excessive platelet activation and pathological thrombosis while improving cardiac function and vascular pathological status. This study may provide solid modern scientific evidence supporting the traditional clinical application of TYTZD for SI-GPBS type CHD and offers potential insights into anti-CHD drug development from the novel perspective of the platelet activation network.

In the present clinical cohort, a total of 754 DEPs were identified between patients with CHD and healthy controls, the functions of which were significantly enriched in pathways, such as platelet activation and aggregation. Among these proteins, RhoA, RAP1B, and ITGA2B were recognized as core nodes in the protein–protein interaction network, and their expression was significantly upregulated. This result is highly consistent with the current understanding of thrombotic events in CHD, namely, that sustained platelet activation is one of the core driving factors for the progression of atherosclerosis and occurrence of acute coronary syndrome [18]. Notably, *ITGA2B*, the gene encoding the αIIb subunit of integrin αIIbβ3, acts not only as a terminal effector target regulated by upstream signaling molecules including RhoA and RAP1B, but may also form a positive feedback loop through outside-in signal transduction mediated by αIIbβ3, thereby amplifying and maintaining platelet activation status and thrombus stability [18]. This allows ITGA2B to transcend the role of a simple adhesion receptor and become a key switch in the dynamic regulation of platelet function, making it a highly promising target for therapeutic intervention. ITGA2B is a key regulator of cardiac health in high-altitude residents [19]. It should be noted that predictive analysis based on network pharmacology only provides theoretical hypothesis and preliminary screening, rather than definitive experimental evidence. Such results further imply the potential importance of this target; pathway enrichment analysis suggests that TYTZD may act on processes such as platelet activation, and it also predicts small-molecule active components that could potentially bind to the ITGA2B protein, such as Celabenzine, PGA1, and Prostaglandin B1. These three active components correspond to *Panax ginseng* C.A. Mey. and *Allium macrostemon* Bunge, which have been reported to inhibit platelet activation [20].

To further verify the above clinical findings and clarify the in-depth molecular mechanism, we constructed a SI-GPBS combined APOE^−/−^ mouse model for subsequent in vivo experimental validation. Cardiac function was significantly impaired in the model group, as characterized by decreased EF% and FS%, whereas TYTZD significantly ameliorated cardiac dysfunction in model mice. Atherosclerosis serves as the core pathological basis of CHD, and lipid deposition is a key initiating factor [21]. Oil Red O staining showed that the model group exhibited extensive aortic lipid deposition and large atherosclerotic plaques, whereas treatment with atorvastatin and TYTZD at all doses significantly reduced lipid deposition. Blood lipid profiles revealed elevated levels of TC, TG, and LDL-C in the model group, whereas the aforementioned treatment groups effectively lowered TC, TG, and LDL-C levels. These findings suggest that TYTZD inhibits atherosclerosis by regulating lipid metabolism, thereby alleviating myocardial injury. Myocardial ischemia and hypoxia can induce myocardial fiber disorganization, necrosis, and inflammatory cell infiltration [22]. HE staining demonstrated that TYTZD improved myocardial fiber arrangement and reduced inflammatory infiltration in CHD mice. Elevated myocardial enzyme levels are indicative of cardiomyocyte necrosis, and TYTZD decreased myocardial enzyme levels, including CK, LDH, and CK-MB. Collectively, TYTZD appears to significantly ameliorated cardiac dysfunction, reduced myocardial enzyme levels, improved myocardial pathological damage, alleviated aortic atherosclerotic plaque burden, and regulated dyslipidemia in CHD mice.

Integrated transcriptomic and proteomic analyses identified 26 DEGs and 72 DEPs whose expression was reversed by TYTZD treatment. Bioinformatic analysis revealed that F2RL2, FGA, FGB, PLEK, CTTN, TGFB1I1, and ITGA2B may be key targets mediating the therapeutic effects of TYTZD. KEGG pathway enrichment analysis showed that these targets were mainly enriched in signaling pathways, such as platelet activation, indicating that TYTZD may exert anti-CHD effects by regulating the platelet activation pathway. Platelet activation plays a crucial role in the progression of CHD. Interactions among activated platelets, inflammatory mediators, and exposed, damaged endothelium impair local coronary microcirculation, leading to thrombosis [23,24]. High concentrations of interleukin-6 have been detected in the serum of patients undergoing clinical PCI and in platelet–leukocyte mixtures aspirated from the coronary arteries, indicating that interventional surgery triggers an inflammatory storm in vivo [25,26]. Platelet–inflammation–endothelium interactions, platelet–leukocyte aggregation, and the formation of bridges between leukocytes and endothelial cells mediated by P-selectin activation exacerbate local coronary embolism. Integrin αIIbβ3 is an important membrane protein on platelets that can bind to RGD-containing ligands, such as fibrinogen, fibrin, and vWF, ultimately cross-linking platelets to form compact fibrin–platelet thrombi [27,28,29]. In high-shear environments, such as the coronary arteries, vWF molecules maintain a continuously unfolded state, which is more conducive to platelet surface receptor aggregation and adhesion [30,31]. In addition, vWF acts as an adhesion molecule that enhances the binding of αIIbβ3 to fibrinogen, promoting the formation of larger and more stable platelet aggregates [32]. TYTZD intervention appears to attenuate platelet adhesion by inhibiting ITGA2B and ITGB3 expression in myocardial tissue and reducing vWF expression.

Activation of αIIbβ3 involves a transition of its extracellular domain to a high-affinity conformation, a process termed inside-out signaling. Subsequently, the exposed receptor sites of αIIbβ3 trigger an outside-in positive feedback pathway, leading to irreversible clot stabilization and retraction [27,33,34]. As the core subunit of αIIbβ3, abnormal upregulation of ITGA2B in mice continuously enhances platelet sensitivity to pro-thrombotic stimuli, creating a persistent pro-thrombotic state in coronary microcirculation. F2RL2, FGA, and FGB are involved in the regulation of outside-in signaling. F2RL2 encodes the thrombin receptor, which mediates thrombin-induced platelet activation. Its abnormal activation promotes platelet aggregation and thrombosis, and its expression is upregulated in the myocardial tissue of mice with myocardial infarction [35]. FGA and FGB are key components of fibrinogen, a protein that facilitates platelet aggregation and adhesion and thereby participates in thrombosis. Elevated fibrinogen levels are closely associated with myocardial thrombosis in CHD models [36]. Consistently, the overactivation of F2RL2 and excessive accumulation of FGA and FGB in model mice further amplified platelet aggregation, aggravated fibrin deposition, and ultimately accelerated coronary microthrombus formation and local myocardial hypoperfusion. By contrast, PLEK, CTTN, and TGFB1I1 participate in the regulation of inside-out signaling. PLEK contributes to thrombosis by modulating platelet adhesion and aggregation and can serve as a potential biomarker for carotid atherosclerosis [37]. In mice, the simultaneous downregulation of ITGA2B and F2RL2 by TYTZD effectively blocked the dual inside-out and outside-in signaling cascades, weakened platelet–endothelium crosstalk, and directly reversed the pathological manifestations including aortic lipid deposition, myocardial inflammatory infiltration and microthrombotic occlusion. CTTN and TGFB1I1 are correlated with pathological processes, such as vascular smooth muscle cell proliferation [38]. Therefore, TYTZD may exert anti-CHD effects by targeting F2RL2, FGA, FGB, and other core molecules to inhibit platelet activation, reduce platelet aggregation and thrombosis, and alleviate inflammatory responses, thereby delaying the progression of atherosclerosis and ameliorating myocardial injury. Notably, TGFB1I1 overexpression promotes angiogenesis [38]. TYTZD can concurrently downregulate the protein expression of key components in this signaling network, indicating that it exerts a synergistic multi-target intervention rather than blocking a single pathway. This mode of action may yield a more balanced regulatory effect, it not only effectively suppresses pathological thrombosis but also potentially reduces the bleeding risk associated with potent antiplatelet monotherapies, such as clopidogrel and ticagrelor.

Vascular endothelial injury and dysfunction initiate platelet activation, and vWF is a key mediator of initial platelet adhesion to damaged vascular walls [39]. TYTZD reduced the co-expression of vWF and CD41 in the myocardial tissue of CHD mice. Inhibition of endothelial activation may fundamentally reduce the triggers of the platelet activation cascade [40], complementing its direct inhibitory effect on intrinsic platelet signaling pathways. In addition, morphological analysis of coronary microthrombi via Carstairs staining provided direct evidence that TYTZD intervention alters the internal composition of myocardial thrombi in CHD mice, reduces dense fibrin–platelet aggregates, and inhibits thrombosis. This morphological observation is consistent with the downregulated expression of integrin αIIbβ3 at the molecular level, suggesting that TYTZD may inhibit myocardial thrombosis by suppressing platelet activation in myocardial tissue.

Network pharmacology and molecular docking analyses only serve as preliminary hypothetical screening and theoretical references to explore the potential targets and signaling pathways of TYTZD; they cannot independently confirm the actual molecular binding activity or pharmacological mechanism. Therefore, the underlying mechanism in this study was further validated by integrating omics profiling data and in vivo animal experimental evidence. This study still has several limitations. First, the mouse CHD model cannot fully recapitulate the pathological characteristics of human coronary heart disease. Obvious interspecific differences in gut microbiota composition and metabolic patterns between humans and mice may cause inconsistent responses to oral TYTZD intervention, which limits the direct clinical translation of animal findings. Second, although computational prediction was performed, in vitro experimental verification of the direct binding affinity between TYTZD active ingredients and core molecular targets is still lacking. Third, this study lacks quantitative analysis of the main chemical components of TYTZD, which weakens the interpretability of the correlation between effective substances and pharmacological activity, and cannot fully guarantee the reproducibility and citability of the research results. Fourth, only the short-term therapeutic efficacy of TYTZD was evaluated in the present work, while its long-term efficacy and safety profile remain to be clarified. In addition, the relatively small sample size of clinical proteomics may weaken the generalizability of the relevant results. In this study, clinical samples were applied to observe phenotypic characteristics, and mouse models were used for mechanistic exploration. Further translational research, quantitative component detection, and well-designed in vitro validation experiments will be conducted in the future to complement and solidify our current findings.

## 4. Methods

### 4.1. Clinical Trial

This study was designed as a prospective case–control study and was approved by the Medical Ethics Committee of the Institute of Basic Theory for Chinese Medicine, China Academy of Chinese Medical Sciences (Approval No.: 2024EC-KY-031, Approved on 29 July 2024). The clinical trial was registered in the International Traditional Medicine Clinical Trial Registry (http://itmctr.ccebtcm.org.cn/). The trial registration number is ITMCTR2025000837, and the registration date was 10 April 2025. All participants provided written informed consent prior to enrollment, and the study protocol was conducted in accordance with the Declaration of Helsinki and relevant clinical research standards. This study was reported in accordance with the TREND 22-item checklist (Table S1), which is provided as a Supplementary File.

### 4.2. Inclusion and Exclusion Criteria

The inclusion criteria for study participants were defined with reference to the 2018 edition of the Guidelines for the Diagnosis and Treatment of Stable Coronary Artery Disease issued by the Cardiology Branch of the Chinese Medical Association: (1) coronary angiography-confirmed stenosis ≥ 50% in at least one major coronary artery branch; (2) age 30–69 years, regardless of sex; (3) no severe cardiovascular events (including acute myocardial infarction, heart failure, or severe arrhythmia) within the past 3 months; and (4) voluntary participation in the study and willingness to cooperate with sample collection and clinical data documentation.

The healthy control group included individuals who underwent routine health checkups at Dongzhimen Hospital, Beijing University of Chinese Medicine, during the same period, with the following criteria: (1) no history of cardiovascular disease or other chronic illnesses; (2) age- and sex-matched with the CHD group; (3) normal results on physical examination, electrocardiogram, liver and kidney function tests, and blood lipid profiles; (4) no unhealthy lifestyle habits, such as smoking or alcohol consumption; and (5) voluntary participation with signed informed consent.

The exclusion criteria were as follows: (1) comorbidities with severe liver or kidney insufficiency, malignant tumors, autoimmune diseases, or hematological disorders; (2) a history of major surgery, trauma, or infectious diseases within the past 6 months; (3) long-term use of glucocorticoids, immunosuppressants, or other drugs that may interfere with protein expression; (4) pregnancy or lactation; and (5) incomplete clinical data or inability to comply with the study protocol.

**Figure 9:**
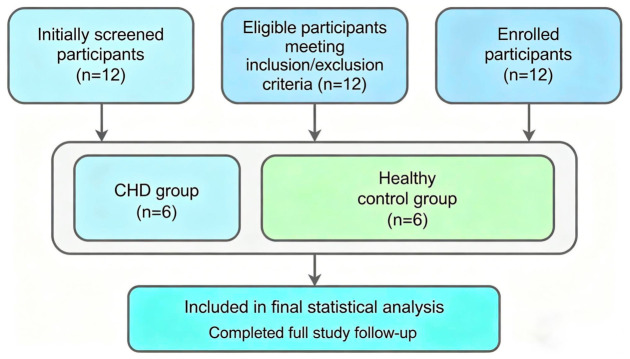
Flow diagram of the clinical trial enrollment and study protocol.

Based on the aforementioned inclusion and exclusion criteria, six patients with CHD who attended the Department of Cardiology, Dongzhimen Hospital, Beijing University of Chinese Medicine, from January 2025 to June 2025 were consecutively enrolled in this study, and six age- and sex-matched healthy control participants were recruited during the same period (Figure 9).

### 4.3. Clinical Sample Collection and Plasma Astral Proteomics Detection

Peripheral venous blood was collected from patients with CHD and healthy controls using EDTA anticoagulation after 12 h of fasting. Plasma was separated by centrifugation at 5000 r·min^−1^ for 15 min at 4 °C (centrifugal radius, 9.9 cm) and stored at −80 °C until analysis. High-abundance plasma proteins were depleted following the manufacturer’s instructions for the Pierce™ Top 14 Abundant Protein Depletion Spin Columns Kit, and protein concentration was determined using the BSA method. For data-independent acquisition (DIA) analysis, chromatographic separation was performed using a nanoflow-rate Vanquish Neo system, and the separated samples were subjected to DIA mass spectrometry using an Astral high-resolution mass spectrometer. The detection parameters were set as follows: positive ion mode; parent ion scan range, *m*/*z* 380–980; full MS resolution, 240,000 at *m*/*z* 200; normalized AGC target, 500%; and maximum injection time, 5 ms. For MS^2^ acquisition, DIA mode was adopted with 299 scan windows, an isolation window of 2 *m*/*z*, a higher-energy collision dissociation collision energy of 25 eV, a normalized AGC target of 500%, and a maximum injection time of 3 ms. DIA data were processed using DIA-NN software. Differentially expressed proteins (DEPs) were screened using the criteria |log_2_^fold change^| > 1 and *p* < 0.05. Functional enrichment analysis of DEPs was performed using the Kyoto Encyclopedia of Genes and Genomes (KEGG) database to explore their biological functions and associated signaling pathways.

### 4.4. Network Pharmacology

Under the criteria of oral bioavailability ≥ 30% and drug-likeness ≥ 0.18, the active components of TYTZD and their corresponding targets were retrieved from the Traditional Chinese Medicine Systems Pharmacology Database and Analysis Platform (https://www.tcmsp-e.com, accessed on 15 November 2025). CHD-related targets were screened from the GeneCards database (https://www.genecards.org/, accessed on 15 November 2025) and Online Mendelian Inheritance in Man database (https://www.omim.org/, accessed on 15 November 2025) using coronary heart disease (CHD) as the search term. Venn diagram analysis was performed to identify the intersection of drug and disease targets, thereby determining the potential active targets of TYTZD for the treatment of CHD. KEGG enrichment analyses were conducted on these targets. Finally, Cytoscape 3.7.1 software was used to construct a drug–component–target network diagram. Molecular docking was performed between the core compounds and targets. The Molecular Operating Environment 2019.0102 software (Chemical Computing Group, Montreal, QC, Canada) was used for energy minimization of the compounds, pretreatment of the target proteins, and identification of active pockets. Subsequently, Molecular Operating Environment 2019 was used to perform molecular docking with 50 iterations. The binding activity between compounds and targets was evaluated based on binding energy, and the results were visualized using PyMOL 2.6.0 and Discovery Studio 2019 software.

### 4.5. Preparation of TYTZD

TYTZD consists of six medicinal herbs with verified botanical names (Table 2): *Panax ginseng* C.A. Mey. (Ginseng Radix et Rhizoma), *Pinellia ternata* (Thunb.) Breit. (Pinelliae Rhizoma, processed), *Trichosanthes kirilowii* Maxim. (Trichosanthis Fructus), *Allium macrostemon* Bunge (Allii Macrostemonis Bulbus), *Paeonia lactiflora* Pall. (Paeoniae Radix Rubra), and *Ligusticum chuanxiong* Hort. (Chuanxiong Rhizoma). All plant names were verified using www.worldfloraonline.org (accessed on 11 December 2025). The aqueous decoction of TYTZD was prepared as follows: 10 g of Ginseng Radix et Rhizoma, 10 g of Pinelliae Rhizoma, 15 g of Trichosanthis Fructus, 15 g of Allii Macrostemonis Bulbus, 15 g of Paeoniae Radix Rubra, and 15 g of Chuanxiong Rhizoma were accurately weighed. All raw medicinal herbs used in TYTZD were purchased from Beijing Sun Tree Kang Pharmaceutical Co., Ltd., Beijing, China, 2407117, 2408023, 2408026, 2407087, 2405099, and 20240104. The botanical identities of all herbal materials was conducted by Director He Xirong of the Institute of Materia Medica, China Academy of Chinese Medical Sciences. For the first decoction, 10-fold the volume of purified water relative to the total weight of the herbs was added, and the mixture was boiled over high heat and then simmered over low heat for 1 h. For the second decoction, eightfold the volume of purified water was added, and the decoction was prepared using the same method as the first decoction. The two decoctions were combined and concentrated to obtain the TYTZD aqueous decoction, which was stored at 4 °C for later use.

### 4.6. UHPLC–MS/MS

Quality control was performed using an Agilent 1200 ultra-high-performance liquid chromatography system (Agilent, Santa Clara, CA, USA) equipped with a YMC J’sphere ODS-H80 column (4.6 × 250 mm, 4 µm). The column temperature was maintained at 25 °C, and the flow rate was 1 mL·min^−1^. The mobile phase comprised water containing 0.1% formic acid (phase A) and methanol containing 30% acetonitrile (phase B). The gradient elution program was as follows: 0–3 min, 5% B; 3–10 min, 5–10% B; 10–15 min, 10% B; 15–25 min, 10–60% B; 25–27 min, 60–90% B. The injection volume was 5 µL, and the ultraviolet detection wavelength was 280 nm.

### 4.7. Establishment and Grouping of Animal Models

One hundred male SPF-grade ApoE^−^/^−^ mice (body weight, 20 ± 5 g) were purchased from Beijing Vital River Laboratory Animal Technology Co., Ltd., Beijing, China, (production license number: SCXK (Jing) 2024-0001) and housed in the SPF-grade animal facility of the Institute of Basic Theory for Chinese Medicine, China Academy of Chinese Medical Sciences (laboratory animal license number: SYXK (Jing) 2021-0017). The feeding environment was maintained at a temperature of 22 ± 1 °C and 50–60% relative humidity. This study was approved by the Animal Experiment Ethics Committee of the Institute of Basic Theory for Chinese Medicine, China Academy of Chinese Medical Sciences (approval number: IBTCMCACMS21-2409-08, Approved on 2 July 2024). After 7 d of acclimatization, 100 ApoE^−^/^−^ mice were randomly divided into two groups, the control group (*n* = 15), fed a normal maintenance diet, and the high-fat diet group (*n* = 85), fed a high-fat diet for eight consecutive weeks. At the end of the 8th week, left anterior descending coronary artery ligation was performed in the ApoE^−^/^−^ mice according to previously published methods [4]. Seventy-five successfully modeled mice were then randomly divided into five groups (*n* = 15 per group): model group, metoprolol tartrate group (MT, 1.3 mg·kg^−1^), low-dose TYTZD group (TY-L, 5.2 g·kg^−1^), medium-dose TYTZD group (TY-M, 10.4 g·kg^−1^), and high-dose TYTZD group (TY-H, 20.8 g·kg^−1^). Mice in the control and model groups were gavaged with an equivalent volume of purified water daily for four consecutive weeks (Figure 4A).

### 4.8. Echocardiographic Assessment of Cardiac Function

Before sample collection, cardiac function was evaluated using an ultrahigh-resolution small-animal ultrasound imaging system (Vevo2100, MS-250 probe, 21 MHz). Five mice per group were fasted for 12 h, fixed in a supine position, and chest hair was removed. Left ventricular ejection fraction (EF%), fractional shortening (FS%), left ventricular end-systolic/diastolic diameters (LVESD/LVEDD), and left ventricular end-systolic/diastolic volumes (LVESV/LVEDV) were measured, with all indicators averaged over three cardiac cycles.

### 4.9. Hematoxylin–Eosin (HE) Staining

Cardiac tissues were fixed in 4% paraformaldehyde for 48 h, dehydrated, paraffin-embedded, and sectioned at 5 µm. After baking and dewaxing, HE staining (Wuhan Servicebio Technology Co., Ltd., Beijing, China, G1005) was performed to observe myocardial fibrinoid deposition, collagen proliferation, and inflammatory cell infiltration.

### 4.10. Detection of Blood Lipid and Myocardial Enzyme Levels

Serum samples from six mice per group were analyzed using ELISA to determine blood lipid and myocardial enzyme levels, including Creatine kinase (CK), lactate dehydrogenase (LDH), creatine kinase isoenzyme MB (CK-MB), aspartate aminotransferase (AST), total cholesterol (TC), triglycerides (TG), low-density lipoprotein cholesterol (LDL-C), and high-density lipoprotein cholesterol (HDL-C) kits (Saisiboer (Beijing) Biotechnology Co., Ltd., Beijing, China, MB-5735A, MB-5900A, MB-5947A, MB-5658A, ADS-W-ZF014, ADS-W-ZF013, ADS-W-D012, ADS-W-D011), strictly following the manufacturer’s instructions.

### 4.11. Whole-Blood Transcriptomic Analysis

Four PAXgene-preserved blood samples per group were thawed, total RNA was extracted using TRIzol reagent, and mRNA was enriched using oligo (dT) magnetic beads. After fragmentation, cDNA synthesis, adapter ligation, library enrichment, and quantification were performed using the QuantiFluor dsDNA System. Libraries were sequenced using the Illumina NovaSeq 6000 platform (paired-end 150 bp). Raw data were quality-controlled using fastp, and gene expression was quantified using featureCounts. Differentially expressed genes (DEGs) were screened using |log_2_ ^fold change^| > 1 and *p* < 0.05, followed by KEGG pathway enrichment analysis.

### 4.12. Plasma Astral Proteomics Detection

Plasma samples from six mice per group were subjected to proteomic analysis, as described in the clinical sample detection protocol.

### 4.13. Weighted Gene Co-Expression Network Analysis

Genes with low expression levels were filtered out (retaining the top 70% by mean expression). Weighted gene co-expression network analysis was used to identify co-regulated gene sets: a soft-thresholding power of β = 9 (scale-free topology R^2^ > 0.85) was selected to construct the weighted adjacency matrix and topological overlap matrix. Modules were identified by hierarchical clustering (minimum of 30 genes per module; merge threshold, 0.25). Pearson’s correlation between module eigengenes and treatment groups was calculated to screen for significant modules.

### 4.14. Western Blotting

Total myocardial proteins were extracted using lysis buffer containing protease and phosphatase inhibitors. Protein concentration was determined using the bicinchoninic acid assay. Proteins were separated by 8% SDS-PAGE, transferred to PVDF membranes, blocked with 5% nonfat milk for 1 h, and incubated with primary antibodies at 4 °C overnight, including anti-GAPDH (Proteintech, Wuhan, China, 60004-1-Ig, 1:5000), anti-F2RL2 (Proteintech, China, 84593-1-RR, 1:5000), anti-FGA (Proteintech, China, 20645-1-AP, 1:1000), anti-FGB (Proteintech, China, 16747-1-AP, 1:1000), anti-ITGA2B (Proteintech, China, 24552-1-AP, 1:2000), anti-ITGB3 (Proteintech, China, 83053-6-RR, 1:5000), anti-TGFB1I1 (Proteintech, China, 10565-1-AP, 1:1000), anti-PLEK (Proteintech, China, 12506-1-AP, 1:1000), anti-CTTN (Proteintech, China, 11381-1-AP, 1:5000). After washing with TBST, membranes were incubated with horseradish peroxidase-conjugated secondary antibodies for 1 h at room temperature. Signals were detected using an ECL kit (Proteintech, China, PK10001) and imaged using a Bio-Rad ChemiDoc XRS+. Band gray values were analyzed using ImageJ.

### 4.15. Detection of Coronary Thrombi

Carstairs staining (Genmed Scientific Inc., Torrance, CA, USA, GMS80128.1) was used to observe the morphology and composition of myocardial coronary microthrombi [41], and histopathological changes were analyzed under a light microscope.

### 4.16. Immunofluorescence Staining

Five-micrometer cardiac sections were baked, dewaxed, hydrated, and incubated with vWF (Proteintech, China, 27186-1-AP, 1:100) and CD31 (Proteintech, China, 11265-1-AP, 1:100) antibodies at 4 °C overnight. After washed 3 times in PBS, the corresponding secondary antibody FITC/PE labeled goat anti-rabbit or anti-mouse IgG was added and incubated for 30 min with constant shaking. After incubation, the slides were washed in PBS. DAPI was added for the staining of nuclei by incubated for 10 min. Next, the spontaneous fluorescent quencher solution was incubated and washed. The high-resolution immunofluorescence results were scanned and saved using a confocal microscope (Leica Biosystems, Singapore). The intensity of the fluorescence was detected in the chemiluminescence analyzer and analyzed with ImageJ software (version 1.54p).

### 4.17. Statistical Analysis

The data are presented as the mean ± standard deviation (SD). Student’s *t*-test was used for comparisons between the two groups, and one-way ANOVA was used for three or more groups. All statistical analyses were carried out using GraphPad Prism software (version 7.0, CA, USA). A *p*-value of less than 0.05 was considered statistically significant.

## 5. Conclusions

**Figure 10:**
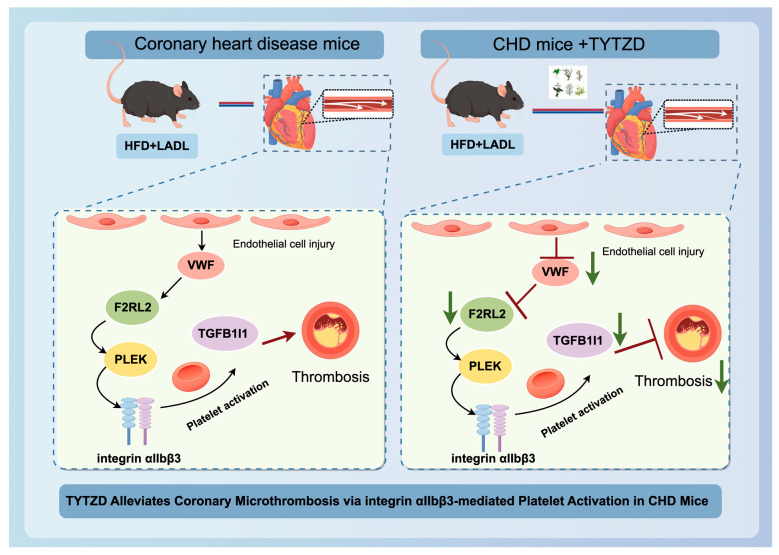
**The schematic diagram of the pharmacological effects and mechanism of TYTZD on CHD with SI-GPBS**.

In conclusion, TYTZD significantly improved cardiac function, reduced myocardial enzyme levels, alleviated myocardial pathological damage, decreased atherosclerotic plaque burden, and effectively regulated dyslipidemia in CHD mice. Mechanistically, TYTZD inhibits integrin αIIbβ3-mediated platelet activation by targeting core molecules, including ITGA2B and F2RL2, thereby blocking coronary thrombosis in CHD mice (Figure 10). These findings suggest that TYTZD holds promise as a potential therapeutic strategy for the clinical management of CHD, and further validate the TCM theory of treating CHD based on resolving phlegm and removing blood stasis.

## Figures and Tables

**Table 1:** The 6 compounds in TYTZD.

No.	Name	Formula	Molecular Weight (Da)	RT (min)
1	Paeoniflorin	C_23_H_28_O_11_	480.16	3.80
2	Ferulate	C_10_H_10_O_4_	194.06	4.03
3	Ginsenoside Re	C_48_H_82_O_18_	946.55	4.21
4	Ginsenoside Rg1	C_42_H_72_O_14_	800.49	4.41
5	Ginsenoside Rb1	C_54_H_92_O_23_	1108.60	5.42
6	Neoligustilide	C_12_H_14_O_2_	190.10	7.65

**Table 2:** Components of TYTZD.

Chinese Name	Latin Name	Medicinal Part	Grams (g)
Ren shen	*Panax ginseng* C. A. Mey.	Root	10
Qing ban xia	*Pinellia ternata* (Thunb.) Breit.	Tuber	10
Gua lou	*Trichosanthes kirilowii* Maxim.	Fruit	15
Xie bai	*Allium macrostemon* Bge.	Bulb	15
Chi shao	*Paeonia lactiflora* Pall.	Root	15
Chuan xiong	*Ligusticum chuanxiong* Hort.	Rhizome	15

## Data Availability

The original contributions presented in this study are included in the article and Supplementary Materials. Further inquiries can be directed to the corresponding authors.

## Supplementary Materials

The following supporting information can be downloaded at: https://www.mdpi.com/article/10.3390/ph19060823/s1, Table S1: the TREND 22-item checklist.

## Abbreviations

CHD	coronary heart disease
SI-GPBS	syndrome of intermingled phlegm and blood stasis
DEGs	differentially expressed genes
DEPs	differentially expressed proteins
DIA	data-independent acquisition
EF	ejection fraction
FS	fractional shortening
HE	hematoxylin–eosin
KEGG	Kyoto Encyclopedia of Genes and Genomes
MT	metoprolol tartrate
TCM	traditional Chinese medicine
TYTZD	Tanyu Tongzhi Decoction
vWF	von Willebrand factor
LDL-C	low-density lipoprotein cholesterol
HDL-C	high-density lipoprotein cholesterol
TC	total cholesterol
TG	triglycerides
CK	creatine kinase
LDH	lactate dehydrogenase
CK-MB	creatine kinase isoenzyme MB
AST	aspartate aminotransferase
LVESD	left ventricular end-systolic diameter
LVEDD	left ventricular end-diastolic diameter
LVESV	left ventricular end-systolic volume
LVEDV	left ventricular end-diastolic volume
PPI	protein–protein interaction
PCA	principal component analysis
WGCNA	Weighted Gene Co-expression Network Analysis
